# Quality Maintenance of Beef Burger Patties by Direct Addiction or Encapsulation of a Prickly Pear Fruit Extract

**DOI:** 10.3389/fmicb.2019.01760

**Published:** 2019-08-09

**Authors:** Lucia Parafati, Rosa Palmeri, Daniela Trippa, Cristina Restuccia, Biagio Fallico

**Affiliations:** Di3A – Dipartimento di Agricoltura, Alimentazione e Ambiente, University of Catania, Catania, Italy

**Keywords:** prickly pear extract, minced meat, natural preservative, health, quality maintenance

## Abstract

Beef burger patties are a very perishable food product with a maximum shelf life of 3 days at 4°C, due to a fast decrease of quality parameters and microbial growth. Although some additives listed in the Regulation EU 601 (2014) are allowed in fresh minced beef and meat preparations with antioxidant functionality, no additive with antimicrobial activity is permitted. In this study, a prickly pear extract (PPE) was added to beef burger patty formulations both by direct application and encapsulation in alginate beads. Beef burger patties were evaluated during refrigerated storage (up to 8 days at 4°C) in terms of microbial quality, pH, texture, and color variation. At the end of storage, burger samples incorporating PPE and encapsulated PPE showed significantly (*p* < 0.05) lower values of mesophilic bacteria, *Enterobacteriaceae*, and *Pseudomonas* spp. when compared to control samples added with sterile distilled water (SDW) or encapsulated SDW. Samples added with encapsulated PPE showed the smallest variations of color a* values (red) during the considered storage period, followed by samples added with PPE, suggesting a protective effect of the extract toward the myoglobin oxidation process. In addition, textural parameters (hardness, cohesiveness, and springiness) reached the highest levels, after 8 days of storage, in burger samples added both with PPE and encapsulated PPE, supporting the potentiality of PPE, encapsulated or not into alginate beads, to be used as a natural preservative of beef burger patty formulations for maintaining quality parameters.

## Introduction

Fresh minced beef is widely used all over the world as a basic ingredient of various food preparations and especially for burger patty formulations. Upon purchase, minced beef should be immediately refrigerated or frozen to avoid the bacterial spoilage. In fact, the grinding process causes the spillage of tissue fluids that represent a rich nutritional component for a wide range of microorganisms, promoting a rapid microbial growth, also if meat is immediately packaged and chilled (Djordjević et al., 2018). The minced beef should be stored at 4°C, for maximum of 3 days, in order to preserve freshness and slow down microbial growth. In addition to worsening of microbiological parameters, prolonged storage leads to a progressive decrease of quality parameters, such as texture and color (Olivera et al., 2013).

Being a very perishable product, food additives can be added during the preparation of minced meat products as reported in the Reg. EU 601 (2014). Some of them, such as alginates (E 401–404), carrageenan (E 407), locust bean gum (E 410), and guar gum (E 412), are added in meat preparations as stabilizers, to reduce water leakage in the packaging and to prevent the loss of meat juices during further processing. Others, such as acetic acid (E 260), potassium acetate (E 261), sodium acetate (E 262), ascorbic acid (E 300), citric acid (E 330), and so on, are allowed as acidulants or antioxidants in pre-packed preparations of fresh minced meat and meat preparations, to which other ingredients than additives or salt have been added, to avoid the decline of the principal quality parameters (Sofos et al., 2013; Reg. EU 601, 2014). In addition, The General Standard for Food Additives (GSFA) of the Codex Alimentarius Commission reports, for non-heat processed meat in whole pieces or cuts (Food Category 08.2.1), among other additives, three natural extracts used as colorants: carmines (INS 120), β-carotenes vegetables [INS 160e (ii)], and grape skin extract [INS 163 (ii)]; as regard to β-carotenes and grape skin extracts, they can be used up to 5,000 mg/kg of meat product (GSFA, 2018).

To date, no additive with antimicrobial activity is allowed on fresh minced meat and meat preparations, but several studies investigated the use of natural antioxidants for meat preservation through the reduction both of microbial growth and lipid oxidation during storage; their involvement in maintaining quality parameters and in improving health benefits has also been studied (Tang et al., 2006; García et al., 2009; Velasco and Williams, 2011; Kumar et al., 2015; Nowak et al., 2016).

Prickly pear extract (PPE) has attracted scientific interest for its healthy properties, including anti-ulcer, anti-inflammatory, diuretic, cardioprotective, and anti-diabetic effects (Feugang et al., 2006; Halmi et al., 2012) and for the low cost of the raw material. With specific regard to food application as a natural preservative, recent research carried out by Palmeri et al. (2018) demonstrated *in vitro* and *in vivo* the great antibacterial activity of a first-crop PPE against pathogenic and spoilage bacteria, generally involved in the decay of sliced beef stored at 4°C; moreover, the extract addition preserved both the beef color and texture over the considered storage period.

With minced beef, being a much more perishable product than the sliced one, the antioxidant/antimicrobial application mode can play a decisive role in preserving the main characteristics of acceptability. Encapsulation of active food ingredients has increased in the food industry since the encapsulated materials can be protected from moisture and oxygen, thus enhancing their stability over time. Özvural et al. (2016) compared the effects of green tea extract (GTE) added with different techniques (direct addition, edible coating, and encapsulation) on quality (particularly oxidative) and microbiological properties of hamburger patties. Their results demonstrated that hamburger treatments by adding or coating with encapsulated GTE solution led to a reduction of lipid oxidation and inhibition of the total mesophilic aerobic microorganisms during storage.

The aim of the present study was to maintain the overall quality of beef burger patties through the incorporation of PPE with two different techniques: direct application or encapsulation in sodium alginate. Beef burger patties were evaluated during refrigerated storage at 4°C in terms of microbial quality, pH, texture, and color variation.

## Materials and Methods

### Preparation of Prickly Pear Extract

First-crop red-purple fruits have been picked in July 2017, gently brushed to eliminate thorns and transported to the Di3A laboratory within 1 h into plastic trays at room temperature. Fruits were washed with tap water and manually peeled. The pulp was lyophilized and the water extract was obtained as described by Palmeri et al. (2018). The red-colored final extract, which had a content of antioxidant pigments betacyanin and betaxanthin of 0.90 ± 0.04 (mg/100 g of pulp) and 0.50 ± 0.01 (mg/100 g of pulp), respectively, was diluted with an equal volume (1:2) of sterile distilled water (SDW) and used for subsequent direct addition or encapsulation on minced beef.

### Encapsulation of Prickly Pear Extract

PPE encapsulation in sodium alginate (Carlo Erba, Cornaredo, MI, Italy) was performed as described by Anbinder et al. (2011) with slight modifications. Briefly, a known volume of diluted PPE, prepared according to the procedure reported above, was mixed with 0.5% (w/v) of sodium alginate and let to stir to homogenize the solution. Once homogenized, the weight of PPE-alginate solution was recorded and afterwards the solution was dropped with a syringe (0.80 mm × 25 mm) into a 1.5% (w/v) calcium chloride solution. The beads formed were filtered through a sterile Whatman^®^ paper Grade 1, allowed to stabilize in the air for 15 min and then weighed to exclude any PPE loss during the spherification process. Control beads were obtained replacing the PPE with SDW.

Alginate bead size, containing SDW or PPE, was estimated using the free license software ImageJ^1^ through the analysis of bead digital images (captured by Scanner Brother MFC-7360N) as reported by Aguirre Calvo and Santagapita (2016). Size of beads was expressed as average Feret’s diameter, corresponding to the longest distance (mm) between any two points along the bead boundary, ± standard deviation. Forty beads for each thesis were analyzed by applying the “analyze particle” command of the software. Alginate beads containing SDW or PPE had a mean Feret’s diameter of 2.15 ± 0.21 and 2.48 ± 0.19 mm, respectively.

### Beef Burger Patty Preparation

Two independent lots of minced meat (2 kg × 1 kg) were purchased from a local supermarket in Catania (Italy), transferred (within 30 min) to the Di3A laboratory in a portable refrigerator at 4 ± 1°C and immediately used for the subsequent meat formulations.

Burger patties were prepared by mixing each lot (1 kg) of the minced beef with 0.8% (w/w) of NaCl in a bowl for 3 min to obtain a uniform mixture, which was divided into four 250 g experimental units; each unit was added with SDW (5%, v/w), encapsulated SDW (5%, v/w), PPE (5%, v/w), or encapsulated PPE (5%, v/w). PPE concentration at 5% (v/w) was adopted on the basis of a previous study carried out on sliced beef (Palmeri et al., 2018), where different concentrations of PPE were tested to select the one that more effectively reduced microbial growth during refrigerated storage. Burger samples from each experimental unit were prepared using a burger press patty maker in order to obtain a standardized size and weight (approximately 40 g, with 6 cm diameter, and 1 cm thickness) and coded as reported in Table 1. Each burger sample was packed under aerobic conditions in a plastic food tray sealed with polyethylene film and stored at 4 ± 1°C in a domestic refrigerator. Burger samples prepared adding SDW, either encapsulated in sodium alginate or not, to the minced beef were used as controls. Each sample of each lot was analyzed for microbial parameters, pH values, color attributes (CIE L*a*b*), and texture after 0, 2, 4, and 8 days of storage.

**Table 1:** Formulations of beef burger patties under study.

	Burger patties’ ingredients
SDW	Minced meat + 0.8% (w/w) NaCl + 5% (v/w) SDW
Encaps-SDW	Minced meat + 0.8% (w/w) NaCl + 5% (v/w) of SDW encapsulated in alginate beads
PPE	Minced meat + 0.8% (w/w) NaCl + 5% (v/w) PPE
Encaps-PPE	Minced meat + 0.8% (w/w) NaCl + 5% (v/w) PPE encapsulated in alginate beads

### Microbiological Analysis

The potential antimicrobial activity of PPE added to the formulation of burger patties, either encapsulated or not in sodium alginate beads, was evaluated by monitoring the microbial population growth after 0, 2, 4, and 8 days of storage at 4 ± 1°C. In brief, a portion of each sample (10 g) was aseptically transferred into a stomacher filter bag containing 90 ml of sterile Ringers solution, homogenized for 5 min and afterward serially diluted. Appropriate dilutions were then plated in Petri dishes containing Plate Count Agar (PCA; Oxoid, Basingstoke, UK) with cycloheximide 0.1% solution (Oxoid), Violet Red Bile Glucose Agar (VRBGA; Oxoid) and Pseudomonas Agar Base (PAB, CM0559, Oxoid), supplemented with Pseudomonas CFC selective agar supplement (SR0103, Oxoid), to monitor the growth of Total Mesophilic Bacteria (TMB), total *Enterobacteriaceae* and total *Pseudomonas* spp., respectively. The plates were incubated for 24–48 h at 32 or 25°C (for the *Pseudomonas* spp. count). Bacterial colonies were counted from three replicates, and the mean was expressed as log CFU (colony forming unit)/g of hamburger ± standard deviation.

### Physical and Chemical Analysis: pH, Color Parameters, and Texture

The pH variation of either treated or untreated beef burgers (Table 1) was monitored during the whole storage. The analysis was carried out at 0, 2, 4, and 8 days by homogenizing 10 g of each sample in 100 ml of distilled water through the Ultra Turrax T18 equipment (IKA ULTRA-TURRAX^®^, Wilmington, NC, USA). Immediately after the sample homogenization, pH was measured by using a Eutech pH 700 Meter (Thermo Fisher Scientific Inc., Waltham, MA, USA). Each value was expressed as mean ± standard deviation of three replicates.

The color of all samples (Table 1) was described in terms of Lightness (L*), redness (a*), and yellowness (b*) space values (CIE L*a*b*). The measurements were carried out on the burger patty surface exposed to air by using a Konica Minolta CM-2500d (Konica Minolta sensing Europe B.V., Bremen, Germany). Color variation was monitored up to 8 days of refrigerated storage and expressed as mean value ± standard deviation of six random readings.

The different burger patties (Table 1) were analyzed for their textural properties by using the Texture Analyzer Zwick/Roell model Z010 (Zwick Roell Italia S.r.l., Genova, Italy) equipped with an aluminum rectangular probe (5 cm × 4 cm). Texture analysis was carried out following the method reported by García et al. (2009) with slight modifications. Each sample, placed between two parallel plates, was compressed to 30% of its original height. Test conditions were: pre-load of 0.01 N, cell load of 50 N, and a cross head speed constant of 50 mm/min. The parameters hardness (N), springiness (cm) and cohesiveness (ratio), representing respectively, the maximum force (Fmax) required to reach the point of break, the ability of the sample to recover its original form, and the degree to which the sample can be deformed before its ruptures, were monitored after 0, 2, 4, and 8 days of storage. Results recorded were expressed as the mean ± standard deviation of three replicates obtained using one hamburger for each measurement.

### Statistical Analysis

Statistical analyses were performed using the Statistical package software Minitab™ version 16.0. All data from experiments were expressed as mean values ± standard deviation. Data of different assays were analyzed independently and subjected to One-way analysis of variance (ANOVA). Fisher’s test was used to compare the significance of differences among means (*p* < 0.05).

## Results

### Microbiological Analysis

**Figure 1:**
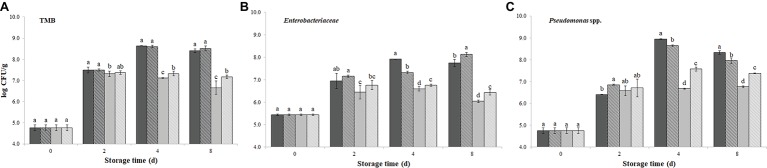
Growth of total mesophilic bacteria (TMB) **(A)**, *Enterobacteriaceae* **(B)**, and *Pseudomonas* spp. **(C)**, evaluated up to 8 days of storage at 4 ± 1°C, on burger patties incorporating prickly pear extract (PPE) and encapsulated prickly pear extract (Encaps-PPE). Controls were made incorporating sterile distilled water (SDW) or encapsulated sterile distilled water (Encaps-SDW). Columns at the same storage time (0, 2, 4, and 8 days) followed by different letters are significantly different according to Fisher’s least significant difference test (*p* < 0.05). Vertical bars indicate the standard deviation of the mean. 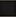 SDW 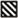 Encapsulated-SDW 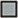 PPE 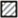 Encapsulated-PPE.

Figures 1A–C displays the microbial counts determined over 8 days of storage (4 ± 1°C) on burger patties with or without PPE, either encapsulated or not in sodium alginate.

The initial TMB value evaluated on PCA was 4.8 log CFU/g in all samples (both PPE and SDW samples) and it rapidly increased after 2 days of storage, exceeding the limit of 6.7 log CFU/g (5 × 10^6^ CFU/g) fixed by the European regulation (Reg. EC 2073, 2005) for total aerobic colony count. After 4 days of storage, while TMB count in control samples SDW and Encaps-SDW further increased, it remained rather unchanged in PPE and Encaps-PPE samples. This different trend appeared more marked at the end of storage (8 days), when TMB count reached the highest values of 8.41 ± 0.08 and 8.51 ± 0.11 log CFU/g in control samples SDW and Encaps-SDW, respectively. Rather, Encaps-PPE and PPE samples showed the lowest values (*p* < 0.05) with 7.17 ± 0.09 and 6.66 ± 0.32 log CFU/g, respectively (Figure 1A), the latter even in compliance with the above-mentioned microbiological limit.

Immediately after treatments (0 days), the amount of *Enterobacteriaceae*, indicating the hygienic condition of raw meat, on burger patties PPE, Encaps-PPE and Encaps-SDW, was not significantly (*p* > 0.05) different from the SDW sample (5.45 ± 0.04 log CFU/g). After 2, 4, and 8 days of storage, the enterobacteria counts registered the lowest values (*p* < 0.05) in samples PPE and Encaps-PPE compared to SDW and Encaps-SDW samples. Although some differences among samples were detected, the addition of PPE strongly inhibited the growth of enterobacteria, registering at the end of the storage (8 days) values averagely 2 log units lower in samples PPE and Encaps-PPE (6.04 ± 0.13 and 6.44 ± 0.09 log CFU/g, respectively) in comparison to SDW and Encaps-SDW samples (7.74 ± 0.16 and 8.13 ± 0.05 log CFU/g, respectively; Figure 1B).

*Pseudomonas* spp. counts (Figure 1C) started from 4.75 ± 0.13 log CFU/g and, over 8 days of storage, increased in all samples, but with considerable differences depending on treatment and sampling time. In particular, after 4 and 8 days all the samples added with PPE displayed significantly (*p* < 0.05) lower values compared to SDW and Encaps-SDW that registered the highest values of 8.94 ± 0.02 and 8.33 ± 0.09 log CFU/g, respectively. At the end of storage (8 days) the best results were obtained by the direct addition of PPE (6.76 ± 0.04 log CFU/g) followed by Encaps-PPE (7.38 ± 0.02 log CFU/g) and Encaps-SDW (7.96 ± 0.12 log CFU/g; Figure 1C).

### pH Determination

Table 2 displays the pH values of burger patties incorporating PPE, either encapsulated or not in sodium alginate, and of control samples (SDW or Encaps-SDW) up to 8 days of storage (4 ± 1°C).

**Table 2:** pH trends of beef burger patties incorporating prickly pear extract (PPE) or sterile distilled water (SDW) over 8 days of storage at 4 ± 1°C.

	Sample			
Storage time (days)	SDW	Encaps-PPE	PPE	Encaps-PPE
0	5.92 ± 0.03^b^	5.94 ± 0.02^b^	6.07 ± 0.06^a^	5.93 ± 0.01^b^
2	5.71 ± 0.02^a^	5.71 ± 0.01^a^	5.35 ± 0.03^b^	5.27 ± 0.02^c^
4	6.45 ± 0.02^a^	5.96 ± 0.01^b^	5.08 ± 0.02^d^	5.17 ± 0.01^c^
8	7.20 ± 0.01^a^	7.06 ± 0.05^b^	5.21 ± 0.01^d^	5.42 ± 0.00^c^

*Data presented as mean ± standard deviation. In each row, values followed by different letter within the same storage time (0, 2, 4, and 8 days) are significantly different according to Fisher’s least significant difference test (p ≤ 0.05)*.

Immediately after treatment (time 0), the samples SDW, Encaps-SDW and Encaps-PPE displayed similar starting pH values of 5.92 ± 0.03, 5.94 ± 0.02, 6.07 ± 0.06, and 5.93 ± 0.01 for SDW, Encaps-SDW, PPE and Encaps-PPE, respectively; sample PPE showed a significantly (*p* < 0.05) higher pH value of 6.07 ± 0.06 when compared to the control (SDW). After 2 days of storage, while the SDW and Encaps-SDW control samples showed a constant pH value (about 5.9), the pH value in the PPE and Encaps-PPE samples significantly (p<0.05) decreased, reaching 5.35 ± 0.03 and 5.27 ± 0.02, respectively. After 4 days of storage, pH value strongly increased in the SDW sample (Table 2), reaching 6.45 ± 0.02. It was almost constant (5.96 ± 0.01) in Encaps-SDW, while, at the same time, sample PPE showed the significantly (p<0.05) lowest values of 5.08 ± 0.02, followed by Encaps-PPE at 5.17 ± 0.01. The difference in pH values was even deeper after 8 days of storage. In fact, the samples PPE and Encaps-PPE displayed values of 5.21 ± 0.01 and 5.42 ± 0.00, respectively, while in SDW and Encaps-SDW samples, pH raised considerably reaching 7.20 ± 0.01 and 7.06 ± 0.05, respectively (Table 2).

### Evaluation of Color Parameters

**Figure 2:**
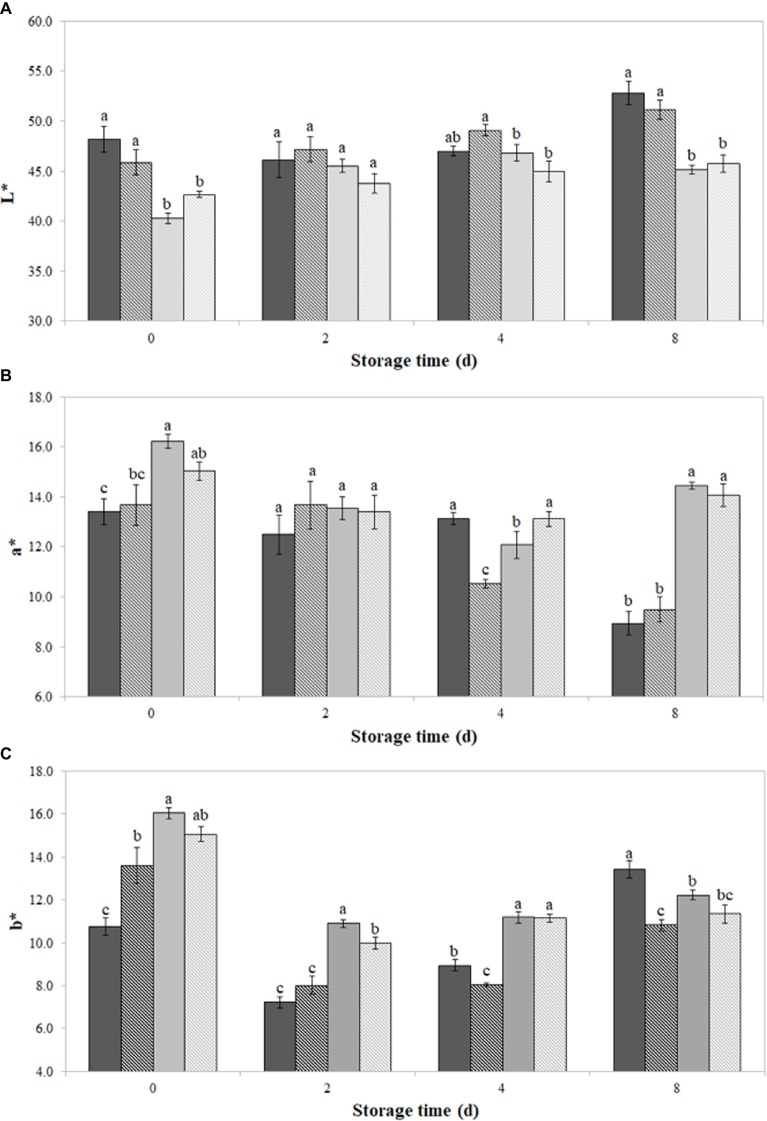
Color values L* **(A)**, a* **(B)**, and b* **(C)** of burger patties incorporating prickly pear extract (PPE) and encapsulated prickly pear extract (Encaps-PPE). Controls were made incorporating sterile distilled water (SDW) or encapsulated sterile distilled water (Encaps-SDW). Color parameters were measured in burger surface up to 8 days of storage at 4 ± 1°C. Vertical bars indicate the standard deviation of the mean. 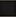 SDW 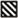 Encapsulated-SDW 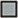 PPE 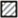 Encapsulated-PPE.

As shown in Figures 2A–C, the incorporation of PPE extract, both direct and encapsulated, significantly (*p* < 0.05) modified the initial L*, a*, and b* values of burger patties, if compared to the respective controls SDW and Encaps-SDW.

In particular, L* showed the lowest (*p* < 0.05) value of 40.3 ± 1.3 when PPE was directly added to minced meat (PPE sample), followed by Encaps-PPE (42.06 ± 0.05) and Encaps-SDW (45.08 ± 0.03) samples. Lightness values of all samples increased after 4 days of storage, recording after 8 days 52.8 ± 1.1, 51.1 ± 0.9, 45.1 ± 0.9, and 45.8 ± 0.4, for SDW, Encaps-SDW, PPE, and Encaps-PPE, respectively (Figure 2A).

As displayed in Figure 2B, burger samples PPE and Encaps-PPE showed initial significantly (*p* < 0.05) higher a* values (16.21 ± 0.8 and 15.03 ± 0.3, respectively) than SDW and Encaps-SDW ones (13.41 ± 0.5 and 13.67 ± 0.4, respectively). After 8 days of storage, SDW and Encaps-SDW samples registered, respectively, a* values of 8.94 ± 0.5 and 9.49 ± 0.4, while PPE and Encaps-PPE samples showed values of 14.45 ± 0.5 and 14.05 ± 0.1. Overall, sample Encaps-PPE showed the lowest variations of a* value during the considered storage period. Sample PPE displayed an irregular trend and registered up to 8 days a decreasing a* value of almost double compared with sample Encaps-PPE (Figure 2B).

The b* values (Figure 2C) detected in samples PPE and Encaps-PPE displayed significantly (*p* < 0.05) higher initial values of 16.04 ± 0.8 and 15.07 ± 0.3, respectively, in comparison to SDW and Encaps-SDW ones, which recorded the values of 10.76 ± 0.4 and 13.60 ± 0.4, respectively. After 2 days of storage, b* values strongly decreased in SDW and Encaps-SDW samples (7.22 ± 0.3 and 8.01 ± 0.3, respectively); at the same time, samples PPE and Encaps-PPE showed a lesser decrease (10.90 ± 0.4 and 9.98 ± 0.2), which can be related to previously observed antimicrobial effect (Figures 1A–C). Overall, Encaps-PPE had a minor influence on the starting color parameters in comparison to PPE; when compared to SDW and Encaps-SDW, samples PPE and Encaps-PPE displayed the most stable trend up to 8 days of storage (Figures 2A–C).

### Texture Analysis

Textural data of burger patties during storage (hardness, springiness and cohesiveness), reported in Table 3, were significantly influenced (*p* < 0.05) by the addition of PPE and Encaps-PPE, according to storage time and the considered parameter.

**Table 3:** Textural properties of beef burger patties incorporating prickly pear extract (PPE) or sterile distilled water (SDW).

Storage time (days)	Textural parameters	Sample			
		SDW	Encaps-SDW	PPE	Encaps-PPE
0	Hardness	10.55 ± 1.46^a^	15.68 ± 4.38^a^	15.74 ± 5.66^a^	14.80 ± 2.90^a^
	Springiness	0.31 ± 0.03^ab^	0.37 ± 0.08^a^	0.29 ± 0.01^b^	0.27 ± 0.00^b^
	Cohesiveness	0.32 ± 0.01^a^	0.26 ± 0.04^b^	0.28 ± 0.01^b^	0.26 ± 0.01^b^
2	Hardness	10.74 ± 2.18^b^	20.17 ± 0.20^a^	20.23 ± 2.02^a^	20.33 ± 4.56^a^
	Springiness	0.33 ± 0.01^a^	0.39 ± 0.11^a^	0.31 ± 0.01^a^	0.29 ± 0.04^a^
	Cohesiveness	0.33 ± 0.01^a^	0.23 ± 0.01^c^	0.30 ± 0.05^ab^	0.27 ± 0.03^b^
4	Hardness	10.11 ± 1.89^b^	20.23 ± 0.17^a^	19.65 ± 1.75^a^	21.65 ± 3.95^a^
	Springiness	0.33 ± 0.01a	0.35 ± 0.10a	0.31 ± 0.01a	0.30 ± 0.03a
	Cohesiveness	0.34 ± 0.00^a^	0.23 ± 0.01^c^	0.31 ± 0.04^ab^	0.28 ± 0.03^b^
8	Hardness	7.46 ± 0.43^c^	8.03 ± 0.86^c^	24.19 ± 4.11^a^	18.70 ± 0.90^b^
	Springiness	0.28 ± 0.00^b^	0.25 ± 0.03^b^	0.37 ± 0.03^a^	0.36 ± 0.02^a^
	Cohesiveness	0.28 ± 0.00^c^	0.29 ± 0.02^c^	0.37 ± 0.02^b^	0.43 ± 0.06^a^

*Data presented as mean ± standard deviation. In each row, values followed by different letter within the same parameter (hardness, springiness, and cohesiveness) are significantly different according to Fisher’s least significant difference test (p ≤ 0.05)*.

In particular, immediately after treatment (time 0), hardness was the same (*p* > 0.05) among treatments (Table 3). After 2 and 4 days of refrigerated storage, all but one, the SDW sample, showed higher hardness values. It reached the highest levels, after 8 days of storage, in sample PPE, followed by Encaps-PPE; no significant difference (*p* > 0.05) was observed between the SDW and Encaps-SDW samples, which registered the lowest hardness values (Table 3). The encapsulation of PPE made this effect less pronounced in sample Encaps-PPE.

During storage, springiness and cohesiveness increased in samples PPE and Encaps-PPE reaching significantly (*p* < 0.05) higher values after 2, 4, and 8 days (Table 3) in comparison to SDW and Encaps-SDW samples.

## Discussion

Microbiological parameters and quality, color, and texture of beef burger patties, prepared both by direct addition or encapsulation in alginate beads of a PPE, were evaluated during refrigerated storage (up to 8 days at 4°C). Microbiological data evidenced at the end of storage a preservative effect both of directly added or encapsulated PPE, which significantly reduced (*p* < 0.05) mesophilic bacteria, *Enterobacteriaceae*, and *Pseudomonas* spp. counts, when compared to control samples added with sterile distilled water (SDW) or encapsulated SDW. However, over storage time, and in particular after 4 days, the direct addiction of PPE to patty formulations seemed to be more effective in limiting the growth of estimated microbial populations; the reasons for this could be attributed to the fact that all the added extract immediately interacted with the bacterial cells, thus reducing their viability, or with beef tissue, thus reducing its degradation with consequent formation of simpler compounds that could be utilized by the microorganisms.

Our results are in accordance with those previously reported by Palmeri et al. (2018) on sliced beef, proving the ability of PPE to effectively reduce the bacterial growth during storage at 4°C. Similarly, Kharrat et al. (2018) reported that the addition of PPE, as a natural preservative, improves the microbiological stability of salami, probably due to the richness of PPE in flavonoids, betalains, and phenolic molecules.

Being the hamburger a very perishable food, both from a microbiological point of view and in terms of the quality characteristics, different alternative strategies have been explored for its preservation. Among them, Özvural et al. (2016) reported as the use of encapsulated green tea extract on burger patty formulation significantly affected the TMB, coliform and yeast and mold count, in comparison to control burger samples. Recently, the same authors investigated the effects of different formulations of chitosan (CS) and chitosan/sodium tripolyphosphate (CS/TPP) matrix solutions including β carotene as additives and edible coatings in hamburger patties, in terms of quality, oxidative and microbiological features; the results showed that incorporation of solution as an edible coating was more effective in lipid oxidation and microbial growth than its utilization as an additive, according to the results on last day (8 days) of storage (Özvural and Huang, 2018).

During storage, the pH values of control samples (SDW and Encaps-SDW) showed an upward trend, probably due to the formation of basic microbial metabolites or metabolites derived from the deaminations of beef proteins (Biswas et al., 2004). The addition of PPE did not substantially change beef pH at the beginning of the storage period considered, so excluding its direct effect on microbial growth. However, samples treated with PPE (both in bulk and encapsulated) showed the lowest pH values during the entire storage period, suggesting an antimicrobial effect of the extract bioactive compounds over time and/or a protective effect of beef tissue and/or a production of organic acids from PPE sugars by heterofermentative microorganisms. By comparing PPE and Encaps-PPE, the last one showed a slightly higher pH value, due to the encapsulation of extract into alginate beads. These results are in accordance with those reported by Palmeri et al. (2018) that evidenced how the pH of stored meat treated with PPE was considerably influenced by different concentrations of the extract.

Referring to color parameters, during storage, control samples evidenced a strongly decreasing trend of red color (a* parameter) probably due to microbial spoilage and consequent increase in pH value to which the color change toward green is typically associated (Borch et al., 1996). Instead, a* values in samples containing PPE, either encapsulated or not, showed a relative stability of this parameter, which suggests a protective effect of the extract toward the myoglobin oxidation process, as already reported by Palmeri et al. (2018). Although there were no significant differences between a* values of samples containing encapsulated or not encapsulated PPE, except at 4 days, samples containing encapsulated PPE showed a more constant trend. Esmer et al. (2011) observed a significant decrease of a* parameter, after the 1^st^ day and during the storage time, of both packaged and fresh minced beef samples. The authors showed also a significant correlation between the a* and b* parameters, due to the formation of metmyoglobin that conduce a decrease of b* value. Control samples (SDW and Encaps-SDW) showed a more pronounced decrease over storage of b* parameter, probably due to oxygen consumption by aerobic microorganisms and to the consequent decrease in oxymyoglobin, which contributes to the formation of the yellow color (Bozkurt, 2006). Also in this case, encapsulation of PPE gave smaller fluctuations of b* parameter over storage time, contributing to the maintenance of color during the storage.

Within textural parameters, at the end of storage (8 days), PPE addition significantly affected hardness, which reached the highest levels in samples added with not encapsulated PPE, probably because of the presence of carbohydrates in the extract (Salim et al., 2009). Similarly, springiness values significantly increased in samples PPE and Encaps-PPE during storage compared to in control samples; the high presences of carbohydrates and soluble proteins in the extract could have caused an improvement in the texture characteristics of the product that acquires higher elasticity and resistance of its structure after the first compression (Kurt and Gençcelep, 2018). Finally, cohesiveness parameter evidenced the highest values in burger patty samples with encapsulated extract, certainly attributable to alginate gelling properties.

## Conclusion

Microbiological control in minced beef has been identified as one of the most important factors in improving quality, extending the shelf life, ensuring product safety and reducing waste.

According to microbiological analysis, the addition of PPE and Encaps-PPE to burger formulation significantly affected the counts of TMB, *Enterobacteriaceae* and *Pseudomonas* spp., up to 8 days of storage, in comparison to control samples. In addition, results showed that, over the storage time, addition of PPE maintained the minced beef at almost constant pH (average 5.3), while the pH of control samples significantly increased, probably due to the production of ammonia, amines and other basic substances by bacterial activity and protein degradation. Although the antimicrobial effect of PPE was slightly more pronounced when it was directly added to burger formulation, the encapsulation of the extract determined more desirable color and texture features. In fact, in terms of color and texture parameters, over 8 days of storage, the sample containing Encaps-PPE showed a more stable trend in comparison to the other treatments.

Certain that the application of PPE extract can be considered as an effective method to contain microbial growth during storage, further studies will be addressed to assess the influence of this extract, encapsulated or not, on the technological characteristics of the cooked product and on its overall sensory acceptability.

The results obtained in this study support the idea of proposing the use of PPE, encapsulated or not into alginate beads, analogously to other extracts (GSFA, 2018) as a natural additive of beef burger patty formulations for maintaining overall quality parameters.

## Data Availability

The raw data supporting the conclusions of this manuscript will be made available by the authors, without undue reservation, to any qualified researcher.

## Author Contributions

RP, CR, and BF conceived and designed the experiments. LP and DT performed the experiments. LP analyzed the data. LP, RP, and CR wrote the manuscript.

### Conflict of Interest Statement

The authors declare that the research was conducted in the absence of any commercial or financial relationships that could be construed as a potential conflict of interest.

## Footnotes

^1^https://imagej.net
